# Self-care behaviours and practices of professional nurses working in primary health care clinics

**DOI:** 10.4102/phcfm.v15i1.4188

**Published:** 2023-11-08

**Authors:** Mukelani L. Muhlare, Charlene Downing

**Affiliations:** 1 Department of Nursing, Faculty of Health Sciences, University of Johannesburg, Johannesburg, South Africa

**Keywords:** behaviour, Orem’s self-care theory, practices, professional nurses, primary health care, self-care

## Abstract

**Background:**

Historically, the nursing profession focused on caring for patients, families and communities but neglected aspects of self-care. Self-care is essential for nurses, as it could impact the quality of care nurses render to patients.

**Aim:**

This article investigated professional nurses’ self-care behaviours and practices in primary health care clinics.

**Setting:**

The study was conducted at selected primary health care facilities (clinics) in regions C and D of the city of Johannesburg, Gauteng.

**Methods:**

A cross-sectional descriptive research survey. Stratified random sampling was used to select respondents. A demographic questionnaire, Self-Care Activities Screening Scale (SASS-14), Nature of Supportive Work Environment questionnaire and Self-Care Work and Home Environmental Factors (SWHEF) questionnaire were combined as the data collection instrument for this study. Descriptive and inferential statistics were used to analyse the collected data.

**Results:**

The finding revealed health consciousness: 86.6% of professional nurses were alert to changes in their health, and 75.2% constantly examined their health. The average professional nurse slept only 7–8 h every day. While 59.4% of professional nurses who participated in the study seldom ate healthy foods (i.e. foods with less sugar, salt, fried snacks or pre-cooked food), 71.7% rarely ate three fruits and two portions of vegetables daily. Only 57.4% of professional nurses regularly drank eight recommended glasses of water daily.

**Conclusion:**

According to the findings, primary health care nurses must prioritise self-care and work in supportive environments.

**Contribution:**

The study acknowledged the need to promote self-care and supportive work environments for professional nurses in primary health care setting.

## Introduction

Younas^1^ defines ‘self-care’ as a critical constituent essential for nurses’ and patients’ well-being. In the same breath, healthcare professionals, particularly professional nurses, have neglected the self-care concept; their focus is primarily on the nurse’s pledge of service, which states that patient care is their priority.^2^ According to Younas,^1^ nurses and nursing students tend to focus on their patients’ self-care and often pay little to no attention to their well-being. However, the best way to care for patients is to care for ourselves as healthcare professionals.^3^ Chipu and Downing concur that quality patient care may be promoted through nurses’ self-care practices.

Self-care is becoming a global concern^4^ and was the focus of the International Centre for Self-Care Research’s inaugural conference in Rome, Italy, in June 2019. The centre’s vision is a world where self-care is prioritised by individuals, families and communities, and is the first line of approach in every healthcare encounter.^5^ Most healthcare workers are passionate nursing professionals. Still, frustration and a sense of powerlessness occur when they cannot provide the needed care to their patients.^6^ Nurses also face similar barriers to self-care as those experienced by the general population. These may include shift work, busy schedules, competing demands for time, cost, availability of resources and work-related stress.^4^ Also, the uncaring clinical learning environment exposes student nurses to stress, fatigue, depression and discourteousness.^7^ Student nurses should ultimately be exposed to self-care behaviours and practices earlier in their nursing career.^1^ Moreover, without a greater understanding of self-care barriers, the outcomes of self-care interventions will remain disappointing.^5^

Despite the challenges nurses face, they try to maintain standards of care and often compromise their health to ensure that patients receive the best healthcare services. During the coronavirus disease 2019 (COVID-19) pandemic, nurses responded exceptionally well to extreme conditions, inconsistent resources and a rapid increase in critical patients in a constricted period.^6^ Nurses’ work-life became of growing interest^4^ considering their increasing workload and insufficient staff complement.

During the pandemic, healthcare professionals worldwide were infected, and some lost their lives while rendering care.^6^ Individuals and nurses wrestled with the pandemic’s new challenges, the shortage of hospital beds, overcrowding in healthcare facilities and nurses’ inability to practise self-care.^8^ Insufficient personal protective equipment and healthcare workers were experienced globally, emphasising the health system’s ill-preparedness for a pandemic.^9^ Nursing professionals have a significant responsibility to care for the ill. Yet, the profession was confronted with moral distress about the pandemic and nurses risking infection while caring for others.^10^ Ultimately, managers and stakeholders should provide nurses with appropriate guidelines based on the availability of resources and safe practices.^10^

Researchers have examined the complexity of self-care and reported that various factors influence nurses’ decisions to engage in self-care practices.^5^ Practising self-care will promote a healing environment that could mitigate the risks of burnout, compassion fatigue and depression and promote resilience and increased patient satisfaction.^11^ The beneficial effects of self-care include improved well-being and lowered morbidity, mortality and reduced healthcare costs.^5^ Nurses affected by COVID-19 trauma-related cases also require education and coping mechanisms to help alleviate the adverse effects on their well-being.^6^ To make recommendations to improve and sustain self-care among professional nurses, the study sought to look into how professional nurses behaved and practised self-care in primary health care clinics.

## Research methods and design

### Study design

A cross-sectional descriptive research survey quantified self-care behaviours and practices among professional nurses.

### Setting

The study was conducted at primary health care clinics in regions C and D of the City of Johannesburg Municipality, Gauteng province. Johannesburg is the largest city in South Africa, also known as ‘Joburg’, which is also one of the most populous urban regions in the nation. The primary health care (PHC) environment in Johannesburg includes various medical facilities and services that meet the demands of a vibrant and culturally varied population. The PHC facilities in the City of Johannesburg provide services to various populations, including those living in urban areas, suburban areas, informal settlements and vulnerable people. In the City of Johannesburg, professional nurses are crucial to providing PHC services. Clinical care, health education, chronic disease management, immunisation programmes and community outreach are among their duties.

### Study population, sample size and sampling strategy

The City of Johannesburg municipality had seven regions: region A (Midrand), region B (Randburg), region C (Roodepoort), region D (Soweto), region E (Sandton), region F (Johannesburg) and region G (Lenasia). Of the seven regions, two were randomly selected, namely region C (11 clinics) and region D (28 clinics). The target population comprised professional nurses employed by the City of Johannesburg. A minimum of 1 year’s working experience was the inclusion criterion for the accessible population. The research study excluded other health professionals, other nursing categories and primary health care clinics not administered by the City of Johannesburg.

The study population was assumed to be 468 professional nurses employed in both areas C and D, with a margin of error of 5.25%, a confidence level of 95%, and an expected response distribution of 50%; the desired sample size was 250, as advised by the statistician.

All the clinic areas C and D were included and the desired sample stratified between facilities on the basis of the monthly headcount. Thus, more nurses were sampled from the large facilities with a higher headcount and more nurses on staff. Systematic random sampling of professional nurses was used at each clinic. The technique employed a beginning point that was randomly chosen and a sampling interval (k) of 2, selecting every second professional nurse on the list until the desired sample size was obtained.

The researcher visited the designated institutions with printed copies of the questionnaire, explaining the study to respondents and emphasising that participation was entirely voluntary and that they were required to sign an accompanying consent form. Every facility had a box for completed questionnaires to be dropped off, and the researcher would visit each facility daily in the afternoon to collect the completed forms.

### Data collection

A demographic questionnaire, Self-Care Activities Screening Scale (SASS-14), Nature of Supportive Work Environment questionnaire and Self-Care Work and Home Environmental Factors (SWHEF) questionnaire were combined as the data collection instrument for this study. Self-Care Activities Screening Scale was created by Elkin Luis Garcia from the University of Navarra, with only the Spanish version receiving validation.^12^ De Kock developed the Nature of Supportive Work Environment questionnaire, validated in South Africa.^13^ The SASS-14 and the Nature of Supportive Work Environment questionnaires did not address the home environmental elements that could impact professional nurses’ ability to practise self-care, so the researcher created the SWHEF for this study. The statistician and the supervisor validated the SWHEF instrument and a pilot study was conducted to test the questionnaire’s practicality. The instruments were amended in response to feedback received from participants in the pilot study, which was acknowledged and considered. The self-administered questionnaire thus comprised three sections: section A focused on respondents’ demographic information. Section B was the SASS-14, which contained a 14-item Likert scale with six response categories ranging from ‘completely disagree’ to ‘completely agree’. Section C presented the Nature of Supportive Work Environment questionnaire, which consisted of an eight-item Likert scale with six response categories ranging from ‘never’ to ‘always’. Section D contained the SWHEF questionnaire, which had an 11-item Likert scale with five response categories ranging from ‘never’ to ‘always’. The research instrument’s reliability was confirmed based on the data from the main study (see Table 1)^14^.

**TABLE 1:** Reliability of the data collection instruments.

Items analysed	Cronbach’s alpha	Cronbach’s alpha based on standardised items	Total number of items
Health consciousness	0.932	0.933	5
Nutrition and physical activity	0.733	0.733	3
Sleep	0.788	0.788	3
Intrapersonal and interpersonal skills	0.680	0.680	3
Supportive work environment: Factor 1 (experienced)	0.861	0.862	4
Supportive work environment: Factor 2 (related factors)	0.741	0.746	3
Self-care work and home environmental factors: Factor 1 (overtime)	0.875	0.976	2

Source: Muhlare, LM. Self-care behaviours and practices of Professional Nurses working in Primary Healthcare Clinics. Johannesburg: University of Johannesburg; 2022. (Unpublished dissertation).

The questionnaires were pilot-tested from 12 December 2021 until 01 January 2022 in region C at three of the City of Johannesburg’s primary healthcare clinics: Weltevredenpark Clinic, Zandspruit Clinic and Princess Clinic. Twenty-five questionnaires were distributed electronically, and 18 were completed and submitted to Google Forms. The sample used for the pilot study was not included in the main study. In response to the pilot study, the researcher adapted question 25 in section D related to SWHEF: ‘Do you use your annual leave?’ This question was replaced with: ‘Do you use your annual leave days for self-care activities, e.g., vacation?’

For the main study, hard copies (questionnaires) were distributed among professional nurses to complete in March 2022. It took approximately 20 min to complete the pilot and main study questionnaires. Because data were collected in primary healthcare facilities, COVID-19 regulations were followed.

### Data analysis

Collected data were entered into an Excel spreadsheet and then into the Statistical Package for the Social Sciences (SPSS) version 26 and analysed. For descriptive statistics, data were summarised; continuous variables such as age, health consciousness, sleep, and intra- and interpersonal skills scores were reported by the mean and median, depending on whether they were normally distributed. For data that were normally distributed, the mean and standard deviation (s.d.) were reported. Frequencies and percentages were calculated based on the number of valid responses for categorical data (with missing values excluded).

Inferential statistics were used to investigate the professional nurses’ responses to their respective ‘self-care activities’. Spearman’s correlation test was used to identify relationships between continuous variables.

### Ethical considerations

Ethical clearance was obtained from the University of Johannesburg, Faculty of Health Sciences Research Ethics Committee (No. REC-1148-2021). The ethical principles to which this study adhered included justice, informed consent, autonomy, beneficence and non-maleficence. All professional nurses received oral and written information and gave informed consent in writing before they could participate in the study.

## Results

Only 209 professional nurses eventually participated in the survey; 41 did not return the questionnaire, and the response rate was 83.6% from all the clinics in regions C and D.

### Demographics

The respondents’ demographic data, measured nominal or ordinal, are displayed in Table 2. Most respondents were female (80.9%), single (42.6%), between the ages of 31 and 40 years (32.5%), held a diploma in nursing (72.2%), had 5 years and longer work experience (87.6%) and were working in the antenatal stream in their respective clinics (31.6%).

**TABLE 2:** Demographic data (*N* = 209).

Variable	Frequency	Percentage
**Age (years)**		
25–30	47	22.5
31–40	68	32.5
41–50	46	22.0
51–60	32	15.3
61–70	14	6.7
71–75	2	1.0
**Gender**		
Male	35	16.7
Female	169	80.9
Do not wish to share	5	2.4
**Work experience (years)**		
Less than 1	1	0.5
1	0	0.0
2	6	2.9
3	8	3.8
4	11	5.3
5 and longer	183	87.6
**Highest qualification**		
Diploma	151	72.2
Bachelor’s degree	28	13.4
Honours degree	24	11.5
Master’s degree	5	2.4
PhD	1	0.5
**Marital status**		
Single	89	42.6
Married	75	35.9
Traditionally married	16	7.7
Cohabiting	3	1.4
Divorced	14	6.7
Widowed	12	5.7
**Work streams in the clinic**		
Anti-natal stream	62	31.6
Child and/or EPI stream	58	29.6
Chronic clinic	56	28.6
Acute clinic	48	24.5
Chest clinic	31	15.8
Emergency	22	11.2
CCMDD	21	10.7

EPI, expanded programme on immunisation; CCMDD, central chronic medicines dispensing and distribution.

### Self-Care Activities Screening Scale

The study found that 66% of professional nurses rarely slept 7h–8 h a day, and a majority (71.7%) did not eat healthy foods or drink the recommended amount of water. In addition, 62.2% of professional nurses needed more time to be more connected to the self. The study revealed that most professional nurses did not learn to do new things, with 75.1% reporting that they ‘never’ did. Similarly, 78.5% of professional nurses did not participate in community initiatives. The study also found that nurses were unlikely to engage in extramural activities after work due to fatigue and a lack of time to rest. Furthermore, 77.6% of professional nurses did not engage in physical activity for at least 30 min daily (Table 3).

**TABLE 3:** Self-Care Activities Screening Scale, Supportive Work Environment and Self-Care Work and Home Environmental Factors mean scores (*N* = 209).

Variable	Mean	s.d.
**Subscale: Health consciousness**		
I am usually aware of my health.	5.0	1.2
I know my inner feelings about my health.	5.0	1.2
I am alert to changes in my health.	4.9	1.3
I reflect on my health.	4.7	1.4
I am constantly examining my health.	4.4	1.4
Total	4.8	1.2
**Subscale: Sleep**		
I sleep 7 h–8 h a day.	4.2	1.5
I think that my rest is of high quality.	4.0	1.6
I find moments to be more connected to myself (I observe, write or reflect on my thoughts, emotions or behaviours).	3.9	1.6
Total	4.0	1.3
**Subscale: Intra- and interpersonal coping skills**		
I am learning to do new things: playing an instrument, sports, practising a new language, cooking, painting, new apps, video games, etc.	3.4	1.6
I actively participate in initiatives in my community (e.g. clapping, singing, playing music, offering my support in what I could help, etc.).	3.0	1.7
I do physical activities (some sports, yoga or dance) for at least 30 min.	3.1	1.6
Total	3.2	1.3
**Subscale: Nutrition and physical activity**		
I eat three servings of fruit and two servings of vegetables daily.	3.5	1.5
I think I am eating better than I used to (less sugar, salt, fried snack or pre-cooked food).	3.9	1.5
I am drinking an average of eight glasses of water a day.	4.0	1.5
Total	3.8	1.2
**Supportive work environment Subscale: Experienced**		
To what degree do you feel valued and respected in your job?	3.5	1.5
To what degree do you receive good support and guidance from your seniors?	3.6	1.5
To what degree do you feel part of a team?	4.1	1.3
To what degree do you experience your workplace as supportive?	3.5	1.5
Total	3.7	1.2
**Subscale: Related factors**		
To what degree do you feel that regular debriefing groups will benefit a supportive working environment?	5.0	1.3
To what degree do you feel a good relationship with your patients will benefit from a supportive work environment?	4.8	1.2
To what degree do you feel open communication with co-workers will benefit a supportive work environment?	4.8	1.2
Total	4.9	1.0
**Self-care work and home environmental factors subscale: Overtime**		
How often are you required to do overtime at work?	2.0	1.1
How often do you do overtime at work?	2.0	1.0
Total	2.0	1.0

s.d., standard deviation.

### Nature of Supportive Work Environment questionnaire

According to the findings, 70.8% of professional nurses did not feel valued and respected in their job, 69% did not receive good support and guidance from their seniors, 59.3% did not feel part of a team and 75.2% did not experience their workplace as supportive. These results suggest that many nurses perceive their work environment as unsupportive and lacking in respect, guidance and teamwork.

### Self-Care Work and Home Environmental Factors

The findings reflected that only a small percentage (1.9%) of professional nurses were frequently required to work overtime; the majority (98.1%) were never needed to work overtime; 91.4% of respondents did not work overtime, while 8.6% did.

### Correlation between health consciousness and self-care dimensions

The correlation coefficient between health consciousness and sleep is *r* = 0.380, which indicates a medium positive correlation between the two variables. This suggests that more health-conscious nurses may also be more likely to prioritise sleep and maintain good sleep habits.

Similarly, the correlation coefficients between health consciousness and intra and interpersonal coping skills, as well as nutrition and physical activity, are also medium and positive, at *r* = 0.382 and *r* = 0.378, respectively. This implies that more health-conscious nurses may also have better coping skills and engage in more healthy eating and exercise habits (see Table 4).

**TABLE 4:** Self-care: Health consciousness correlation with self-care questionnaire dimension (*N* = 209).

Self-care questionnaire dimension	Pearson correlation	Significance
Sleep	*r* = 0.380	*p* < 0.001
Intra- and interpersonal coping skills	*r* = 0.382	*p* < 0.001
Nutrition and physical activity	*r* = 0.378	*p* < 0.001

## Discussion

### Discussion of key findings

The study’s findings reflected the various self-care practices respondents engaged in, which were classified into four dimensions: health consciousness, sleep, intra- and interpersonal coping skills, and nutrition and physical activities. These dimensions encompassed a wide range of self-care activities that respondents reportedly engaged in to care for their physical, emotional and mental well-being.

The results identified two supportive work environment dimensions, which were labelled as ‘factor 1 (experience)’ and ‘factor 2 (related factors)’. The study reported on the SWHEF respondents faced daily. Specifically, the study identified one dimension of such factors: overtime.

### Self-care activities

#### Health consciousness

The findings suggest a positive shift in respondents’ attitudes and behaviour regarding their own health awareness. According to the results, most respondents (86.6%) reported being attentive to changes in their health. This indicates that these nurses actively monitor and pay attention to any potential changes or symptoms that may affect their well-being. This result is noteworthy, especially compared to the previous study conducted by Linton and Koonmen,^15^ which reported that only 32% of the nurses surveyed were alert to changes in their health. The stark contrast between these findings suggests a significant improvement in nurses’ health awareness. It could indicate a growing recognition of the importance of self-care and a greater emphasis on personal health management within the nursing profession.

The study conducted by Nkabinde-Thamae et al.^16^ further supports the significance of nurses’ health awareness. Their research revealed that nurses unaware of changes in their health tended to neglect their self-care. This neglect, in turn, could lead to unfavourable consequences, such as a compromised ability to provide optimal care to patients, families and the community. The current study shows a higher percentage of nurses reporting attentiveness to their health, suggesting a positive shift in attitudes and practices within the nursing profession. Nurses who are actively aware of their health are more likely to prioritise self-care and well-being. By doing so, they can better manage their own health, which ultimately benefits both themselves and the patients under their care.

#### Sleep

The study’s findings suggest a positive trend in respondents’ perceived quality of rest. According to the results, a majority (67.4%) stated they achieved high-quality rest. This indicates that these nurses perceive their restful periods as satisfactory in quantity and quality. The contrast between these findings and the prior study conducted by Silva et al.^17^ is noteworthy. In their research, a majority (64.3%) of surveyed nurses reported having poor-quality rest. The difference in results could suggest a potential improvement in nurses’ rest patterns and perceived quality of rest over time.

#### Intra- and interpersonal coping skills

The results of the current study indicate that a significant majority of professional nurses (75.1%) reported not engaging in learning new skills, such as playing an instrument or sports. This finding suggests a potential lack of opportunities or time constraints for nurses to pursue personal interests or hobbies outside of their professional responsibilities. This aligns with the explanation provided by Linton and Koonmen^14^ that nurses’ exhaustion and limited time to rest make it unlikely for them to participate in extramural activities after work. Furthermore, the study found that a substantial percentage of nurses (78.5%) did not participate in community initiatives, while a smaller proportion (21.5%) reported involvement in community activities. The lack of participation in community initiatives may be attributed to nurses’ demanding work schedules and limited opportunities for engagement outside of their work environment. As McNamara^18^ noted, nurses often experience fatigue and have limited time to rest, making it challenging to allocate time and energy to community activities.

#### Nutrition and physical activity

McNamara’s^18^ and the current study’s findings highlight concerning patterns in professional nurses’ dietary habits. In McNamara’s^18^ study, only 13% of respondents reportedly consumed the recommended five or more servings of fruits and vegetables daily. Similarly, in the current study, most professional nurses (71.7%) reported rarely consuming three servings of fruit and two servings of vegetables daily. These results indicate a significant gap between dietary recommendations and nurses’ actual eating habits, attributed to a potential lack of access to healthy food choices in the work environment. McNamara’s^18^ study reported that 54% of respondents had access to healthy food choices at work. Similarly, the current study found that 59.4% of nurses did not eat healthy foods, such as those with less sugar, salt, fried snacks or pre-cooked meals. These findings indicate that nurses’ work schedules and environments may hinder their ability to make healthy dietary choices while on duty.

### Supportive work environment

#### Experience

The study’s findings highlight issues regarding professional nurses’ experiences in their work environment. According to the results, most nurses reported feeling undervalued and disrespected in their job (70.8%), lacking good support and guidance from their seniors (69%), not feeling part of a team (59.3%) and not experiencing their workplace as supportive (75.2%). These findings align with previous research in this area, emphasising nurses’ persistent challenges in their work environments. Feeling undervalued and disrespected can significantly impact nurses’ job satisfaction, motivation and overall well-being.^19^ When nurses do not receive the recognition and respect they deserve, it can lead to frustration, demotivation and potential burnout.^20^ This, in turn, can negatively affect the quality of care they provide to their patients.

#### Related factors

The study’s findings highlight the importance of specific factors contributing to a supportive work environment for professional nurses. Most nurses in the study believed that regular debriefing sessions (88%), a good relationship with patients (86.6%) and open communication with co-workers (84.7%) would promote a supportive work environment. These findings are consistent with previous research that also emphasised the significance of communication, relationships and debriefing sessions in creating a supportive and positive workplace for nurses.

Regular debriefing sessions allow nurses to reflect on their experiences, discuss challenging cases or situations, and share their thoughts and emotions with colleagues.^21^ These sessions can benefit nurses’ emotional well-being, stress management and resilience. It allows them to process difficult events and receive support from their peers, fostering a sense of camaraderie and shared understanding.^21^ Debriefing sessions also offer a learning and professional development platform, as nurses can exchange knowledge, strategies and best practices.

### Self-care work and home environmental factors

#### Overtime

The findings revealed a notable discrepancy regarding the prevalence of overtime work among professional nurses. The current study reports that most nurses (98.1%) were not required to work overtime, while a small percentage (1.9%) experienced frequent overtime requirements. However, these results contradict those of Silva et al.,^22^ who reported that many nurses were required to work overtime to cover staff shortages. Overtime work practices can vary widely across different countries and healthcare settings due to staffing levels, policies and resource availability variations.^22^ Therefore, nurses’ prevalence of overtime work may differ considerably based on these factors. A study conducted by Bae et al.^20^ (in Cambodia) revealed that more than one-fifth of nurses worked beyond the legal work-hour limit. This finding suggests that overtime work can be a prevalent issue in specific regions and healthcare contexts, potentially adversely affecting nurses’ well-being and patient care.

### Limitations of the study

This study only focused on professional nurses working in primary healthcare clinics in the City of Johannesburg, Gauteng province. The study was limited to professional nurses within the primary health care setting and not professional nurses working in hospitals. The study only focused on professional nurses and no other nursing categories like enrolled nurses or other healthcare professionals like medical practitioners. The study was also limited to the views of professional nurses working in regions C and D of the City of Johannesburg; those working in regions A, B, E, F and G were not considered. The study only focused on the City of Johannesburg clinics and not the other municipalities’ clinics, like those of the City of Ekurhuleni. The instrument developed by the authors could be further validated by additional experts in caring and self-care.

### Recommendations

The recommendations for the City of Johannesburg Health Department, nursing practice, nursing education and nursing research aim to improve primary health care employees’ physical and mental health. The research committee should encourage healthcare professionals to participate in research, while sports programmes and vegetable gardens could promote physical activity and healthy eating. Access to clean drinking water and a self-care policy must also be provided. For nursing practice, providing necessary tools and resources, establishing regular debriefing programmes, encouraging communication among co-workers, and guiding and valuing subordinates will create a supportive work environment. Additionally, strategies to encourage nurses to use tea and lunch breaks and take annual leave and sick days for self-care are recommended. Nursing education should incorporate self-care strategies in the curriculum, while a similar study should be conducted in other regions of the City of Johannesburg and include other nursing categories. Finally, a qualitative study should be conducted to understand nurses’ opinions and feelings regarding self-care, and a self-care policy should be developed for nurses working in primary health care clinics. These recommendations can improve primary health care employees’ physical and mental health and enhance the quality of care provided to patients.

## Conclusion

Using a cross-sectional and comparative descriptive research method enabled the researcher to gain information regarding professional nurses’ self-care practices and work environments in primary health care settings. The study determined a need to promote self-care and supportive work environments for professional nurses working in primary health care settings. Without awareness and the promotion of self-care and supportive work environments, professional nurses will continue to experience a negative perception of themselves and their work environment as they care for their patients, families and communities. The nursing profession needs passionate and caring nurses, but it also needs nurses who will prioritise self-care. Nurses should thus be encouraged to eat healthily, regularly exercise and engage in regular debriefing sessions, among other strategies. It was also determined that, although nurses face several barriers in relation to self-care, some nurses can prioritise self-care practices and still regard their work environment as conducive and supportive.
